# Effects of Bipolar Disorder on the Verbal Fluency Skills of Native Speakers

**DOI:** 10.3390/brainsci16020228

**Published:** 2026-02-14

**Authors:** Bertuğ Sakın, Dilek Eroğlu Uzun, Mehmet Emrah Cangi, Ali Görkem Gençer, Mehtap Arslan, Selman Aktaş

**Affiliations:** 1 Department of Speech and Language Therapy, Hamidiye Faculty of Health Sciences, University of Health Sciences, Istanbul 34660, Türkiye; 2 Department of Speech and Language Therapy, Faculty of Health Sciences, Biruni University, Istanbul 34010, Türkiye; 3 Department of Psychiatry, Haydarpaşa Numune Training and Research Hospital, Istanbul 34660, Türkiye; 4 Department of Biostatistics and Medical Informatics, Hamidiye Faculty of Medicine, University of Health Sciences, Istanbul 34660, Türkiye

**Keywords:** bipolar disorder, language disorders, neurolinguistics, Turkish, verbal fluency skills

## Abstract

Background/Objectives: Bipolar disorder (BD) is a chronic psychiatric condition characterized by episodes of mania, hypomania, and depression. Due to the cognitive impairments associated with BD, patients frequently experience difficulties in attention, memory, and executive functions, which in turn adversely affect specific aspects of their language abilities, such as word retrieval, verbal fluency, and the organization of coherent speech. The present study aims to determine the extent to which the verbal fluency skills of native Turkish-speaking individuals with BD are impaired compared to healthy controls and to identify whether there are differences in verbal fluency skills and their subcategories between bipolar I disorder (BD I) and bipolar II disorder (BD II) groups. Methods: A cross-sectional comparative design was employed in this study, including 39 euthymic patients diagnosed with BD I or BD II and 39 healthy controls. Verbal fluency was assessed using a standardized task comprising semantic fluency, semantic switching, phonemic fluency, and automatic speech subtests. All assessments were conducted under blinded conditions, and scoring was performed by independent raters. Group comparisons were carried out using ANOVA, Kruskal–Wallis, and ANCOVA analyses; age was controlled for through covariance analysis. Additionally, sensitivity analyses were conducted within the 25–55 age range. Results: The control group demonstrated significantly higher performance than the BD groups across all semantic and phonemic verbal fluency tasks. No statistically significant differences were observed between the groups in automatic speech tasks. When comparing the BD I and BD II groups, a statistically significant difference was found only in the action (verb) category, with the BD II group outperforming the BD I group. Conclusions: The findings indicate that bipolar disorder is associated with marked impairments in semantic and phonemic verbal fluency, while automatic speech abilities appear to be relatively preserved. Moreover, the observed difference between BD subtypes—particularly in the action (verb) category—suggests that the type of the disorder may differentially influence cognitive–linguistic functioning.

## 1. Introduction

Bipolar disorder (BD) is a chronic psychiatric condition that is not only highly prevalent in the general population but also leads to significant functional impairment, making early diagnosis and timely initiation of treatment critically important [1]. BD is characterized by unpredictable mood fluctuations whose sequence and duration cannot be foreseen. These mood episodes include periods of mania, hypomania, and depression [2]. According to the DSM-5 [3], mania is defined as a clinical state lasting at least one week (shorter if hospitalization is required), characterized by an abnormally elevated, expansive, or irritable mood, accompanied by at least three of the following symptoms: inflated self-esteem or grandiosity, decreased need for sleep, increased speech rate or talkativeness, flight of ideas, heightened distractibility, increased goal-directed activity or psychomotor agitation, and excessive involvement in risky activities. Mania is associated with marked impairment in social and/or occupational functioning. Hypomania is a clinical state with the same symptoms as mania, lasting for a minimum of four days, but with less severe impairment in social and occupational functioning. In DSM-5, depression is described as a clinical state lasting at least two weeks, characterized by a depressed mood and/or anhedonia, accompanied by at least four additional symptoms: changes in sleep or appetite, psychomotor agitation or retardation, decreased energy, feelings of worthlessness or guilt, diminished concentration, and suicidal ideation. Depression also presents with significant impairment in social and occupational functioning [3].

An individual who has experienced at least one manic episode during their lifetime is diagnosed with bipolar I disorder (BD I), regardless of whether they have experienced a depressive episode. In contrast, a diagnosis of bipolar II disorder (BD II) requires the individual to have experienced at least one hypomanic episode and one depressive episode during their lifetime [3]. Although studies have reported varying findings regarding the lifetime prevalence of BD, research that accounts for subthreshold conditions has estimated rates as high as 5%. The annual incidence rate has been reported as 1.4% [2]. During these episodes, individuals may engage in behaviors that are potentially life-threatening and detrimental to quality of life, such as self-harm, substance use, reckless driving, infidelity, and suicide attempts [4]. Research further demonstrates that depressive symptoms are the most influential in reducing patients’ quality of life and overall functioning. Moreover, longer episode duration and early onset are also associated with adverse effects on these parameters [5].

BD occurs equally in men and women. The average age of onset is between 17 and 25 years, with men experiencing onset approximately five years earlier than women [6]. BD is one of the psychiatric disorders with the highest rate of genetic transmission [7]. A positive family history increases the likelihood of developing the disorder by up to eightfold, while the risk rises by 50–75% if both parents are diagnosed with BD [8]. In addition to genetic factors, imbalances in neurotransmitter systems also play a role in the etiology of BD. Evidence suggests that manic episodes may result from dopaminergic hyperactivity, disturbances in the norepinephrine-acetylcholine balance, and serotonin elevation observed following L-tryptophan administration, which can induce manic states [6,9]. In recent years, increasing research has focused on the role of acetylcholine, GABA, and glutamate in the etiology of BD [10].

Studies have demonstrated that patients with BD exhibit impairments in attention, memory, and executive functions, and that these impairments negatively affect their language abilities. The primary underlying cause of these difficulties is the cognitive deterioration associated with the disorder. Verbal fluency refers to an individual’s ability to generate words belonging to specific categories within a limited period of time. In the literature, when compared with control groups, BD patients have been shown to display alterations in semantic content, deficits in verbal association, and inconsistencies in word production speed and prosody [11,12,13,14,15,16,17].

The aim of this study is to determine the extent to which the verbal fluency skills of BD patients who are native speakers of Turkish are impaired compared to healthy individuals and whether there are differences in verbal fluency performance and its subcategories between BD I and BD II. The hypothesis of the study is that if BD directly affects the cognitive functions that constitute the fundamental infrastructure of language competence in the brain, it will also negatively influence verbal fluency skills, which rely on the integration of a broad cognitive network. Given that clinical, cognitive, and genetic differences exist between BD subtypes [18], it is expected that BD I and BD II patients will differ in their verbal fluency performance and across its subcategories.

## 2. Methodology

### 2.1. Study Design

This study employed a cross-sectional comparative design. The verbal fluency skills of BD patients who are native speakers of Turkish were analyzed and compared with those of a healthy control group. In addition, the study examined the verbal fluency performance of BD I and BD II patients.

### 2.2. Participants

The study sample consisted of 39 BD patients (25 females and 14 males) who applied to the Psychiatry Clinic at Istanbul Haydarpaşa Numune Training and Research Hospital, were diagnosed with BD I or BD II by an expert clinical psychiatrist using structured interviews according to DSM-5 criteria, regularly attended follow-up appointments, were monolingual, and spoke Turkish as their native language. The control group consisted of 39 healthy volunteers (25 females and 14 males).

Participants in the BD group met the following inclusion criteria: a diagnosis of BD I or BD II according to DSM-5 criteria, age between 19 and 66 years, being a native Turkish speaker, regular attendance at follow-up appointments, absence of any other medical conditions that could affect cognitive functions, and no current or prior receipt of speech and language therapy.

All bipolar disorder patients included in the study were in a euthymic mood state at the time of assessment, and no residual symptoms were observed during the evaluation. Euthymic mood and the absence of residual symptoms were confirmed through structured interviews conducted by a board-certified psychiatrist, along with a review of medical records. Individuals exhibiting acute manic, hypomanic, depressive, or residual symptoms were not included in the study. All participants were on a stable medication regimen and were not in a medication adjustment (titration) period at the time of assessment. This approach was implemented to minimize the potential impact of mood fluctuations on cognitive and language performance. All control participants had no history of psychiatric disorders and had never used psychiatric medications. No participant in the study was excluded due to misunderstanding or noncompliance.

Prior to participation, all participants provided informed consent. The study was approved by the local ethics committee and conducted in accordance with the principles of the Helsinki Declaration.

### 2.3. Data Collection Instruments

In the study, the following instruments were used to collect data: a participant information form including demographic data, a voluntary informed consent form, and a verbal fluency task. The verbal fluency task was administered to assess the participants’ verbal fluency abilities.

### 2.4. Verbal Fluency Task

The verbal fluency task used in this study was administered according to the criteria and instructions developed by Tunçer [19] for assessing the verbal fluency skills of Turkish-speaking adults. The task consisted of four distinct sections: semantic fluency, semantic switching, phonemic fluency, and automatic speech. The semantic fluency section included tasks targeting household items, beverages, actions, and emotion words [20]. The semantic switching section involved tasks requiring the sequential production of words in the categories of animals and fruits [21]. The phonemic fluency section was based on the FAS test and required participants to generate words beginning with the letters K, A, and S, which are among the most frequently used sounds in Turkish verbal fluency assessments [22,23]. Finally, the automatic speech section included tasks in which participants were asked to produce sequences such as counting months and days.

To ensure standardized administration, all tasks were presented using Microsoft PowerPoint. At the beginning of the task, a blank screen was displayed for 10,000 ms to allow participants to prepare. Subsequently, instructions for each section were displayed on the screen for 4000 ms. After the instructions, a blue-dotted screen remained for 60,000 ms, during which participants were expected to generate responses according to the instructions. The 60,000 ms task duration is a standard administration period that has been widely used in the assessment of verbal fluency performance in Turkish-speaking samples and for which normative data are available [24,25]. This duration has been reported to adequately sample lexical access and executive control processes while minimizing participant fatigue and ensuring high clinical applicability. In the verbal fluency task, each correct response was scored as 1 point, while incorrect responses that did not belong to the target category and repetitions were scored as 0 (correct words were included, while incorrect words and repetitions were not included). Participants were instructed to produce as many words as possible for all verbal fluency categories and to avoid repeating previously generated words. The task administration and scoring procedures used in the study were conducted in accordance with validated protocols.

### 2.5. Procedure

Data collection was conducted at the Psychiatry Clinic of Istanbul Haydarpaşa Numune Training and Research Hospital for the BD group and at the University of Health Sciences for the control group. BD I and BD II diagnoses were established by a specialist psychiatrist. Participants who met the inclusion criteria and control participants were first given detailed information about the study. A demographic information form was completed to collect participants’ demographic data. Subsequently, the informed consent form was distributed to all participants and signed. Following this, the researchers provided instructions for the verbal fluency task. All participant responses were recorded as audio files and transcribed verbatim without additions or omissions.

### 2.6. Blinding and Inter-Rater Reliability

The researcher administering the verbal fluency tasks was blind to the participants’ diagnostic groups. All responses were audio-recorded and transcribed. The quantitative scoring of the verbal fluency tasks was conducted independently by two experienced research assistants who were blinded to the participants’ diagnostic groups, clinical characteristics, and demographic information. Inter-rater reliability was evaluated using the intraclass correlation coefficient (ICC[2,1]) calculated with a two-way random-effects model and an absolute agreement approach, and the results are presented in Table 1.

Inter-rater reliability for the verbal fluency tasks was assessed using a single-measure, two-way random-effects ICC (ICC[2,1]). Values ranged from 0.854 to 0.915, indicating good to excellent reliability (see Table 1). All participants received standardized instructions prior to the tasks, and all procedures were conducted according to a fixed protocol. This approach minimized potential bias in task administration and scoring.

### 2.7. Data Analysis

All statistical analyses were conducted in R version 4.4.2 (R Foundation for Statistical Computing, Vienna, Austria) within the RStudio 2024.12.0+467 (Kousa Dogwood) environment. Data visualization and fully reproducible reporting were implemented using Quarto (version 1.5.57). Descriptive statistics were calculated for all study variables. Continuous variables were summarized as mean with standard deviation or median with min-max values, depending on their distributional characteristics, while categorical variables were presented as frequencies and percentages.

Prior to group comparisons, the distributional properties of the variables were assessed. For variables meeting the assumptions of normality and homogeneity of variances, one-way analysis of variance (ANOVA) was used to compare differences among the BD-I, BD-II, and control groups. When a significant overall group effect was observed, Bonferroni-adjusted post hoc tests were applied to determine pairwise group differences. Effect sizes for parametric analyses were reported using eta squared (η^2^).

For variables that did not meet parametric assumptions, Kruskal–Wallis tests were conducted to evaluate group differences. In cases of significant Kruskal–Wallis results, Dunn’s post hoc tests with Bonferroni correction were used for multiple comparisons. Effect sizes for non-parametric analyses were quantified using epsilon squared (ε^2^) to reflect the magnitude of between-group differences.

Comparisons of categorical variables across groups were performed using chi-square tests, with effect sizes reported as Cramér’s V where appropriate. All statistical tests were two-tailed, and a *p*-value < 0.05 was considered statistically significant.

To assess whether the sample size was sufficient to detect the observed effects, a post hoc power analysis was conducted using G*Power 3.1 software for one-way ANOVA. Effect sizes were calculated from the observed eta-squared (*η*^2^) values and converted to Cohen’s f values using the standard formula $f=\sqrt{\frac{\eta^{2}}{1-\eta^{2}}}$. The observed effect sizes were large (Cohen’s f = 0.85–1.18), indicating that the statistical power for all primary outcome variables exceeded 0.80. These results demonstrate that the sample size used in the study was adequate to reliably detect differences between groups.

To control for age heterogeneity between the BD-I and BD-II groups, one-way ANCOVA analyses were conducted for each verbal fluency measure, with age included as a covariate. Effect sizes were calculated using partial eta squared (η^2^p) along with 95% confidence intervals (CI). The statistical significance threshold was set at *p* < 0.05. This approach ensures that the observed group differences are independent of age and allows for a transparent reporting of the effect of the diagnostic group on verbal fluency tasks.

Additionally, an age-restricted sensitivity analysis was conducted. In this context, the sample was limited to participants aged 25–55 years, and all analyses were re-run within this subsample. This age range was selected to maintain adequate group sizes while reducing the potential influence of extreme age values in the distribution.

## 3. Results

Age-adjusted ANCOVA analyses indicated that the effect of diagnostic group was statistically significant for the following verbal fluency tasks: Household Items, Beverages, Action Words, Emotion Words, Animals and Fruits, as well as phonemic fluency tasks beginning with the letters /k/, /s/, and /a/ (all *p* < 0.001). Effect sizes were large, with partial eta squared (η^2^p) values ranging from 0.35 to 0.85, and 95% confidence intervals are presented in Table 2. In contrast, no statistically significant effect of diagnostic group was observed for the Months and Days tasks (*p* = 0.207 and *p* = 0.243, respectively), with small effect sizes (η^2^p < 0.05), indicating limited group differences in these domains. Across all models, the effect of age was not significant (all *p* > 0.05), and effect sizes were negligible (η^2^p < 0.02), confirming that the observed group differences were independent of age.

In the age-restricted (25–55 years) sample, between-group differences in verbal fluency tasks were evaluated. In the sensitivity analyses, independent of the primary analyses, a uniform and conservative analytical approach was deliberately adopted for all variables; results were reported as median (minimum–maximum), and between-group comparisons were conducted using the Kruskal–Wallis test. Detailed results of these analyses are presented in Table 3.

The demographic and clinical characteristics of the BD I, BD II, and control groups are presented in Table 4. No statistically significant differences were found among the groups in terms of age, sex distribution, educational level, or socioeconomic status (*p* > 0.05). These findings indicate that the study groups were comparable and homogeneous with respect to the main demographic variables. Moreover, the small effect sizes observed in these comparisons further support the demographic similarity among the groups.

In contrast, marked differences were observed among the groups in verbal fluency skills. Statistically significant differences were found across the groups in semantic fluency tasks such as Household Items, Emotion Words, and Animals and Fruits (all *p* < 0.001). Effect sizes for these variables ranged from moderate to very large, with the highest effect size observed in the Animals and Fruits task (ε^2^ = 0.79). These findings indicate that the differences among the groups are not only statistically significant but also clinically meaningful.

Similarly, significant differences were observed among the groups in phonemic fluency tasks (/k/, /s/, and /a/ sounds) as well as in the Beverages and Action Words categories (all *p* < 0.001). Effect sizes for these variables were large (η^2^ = 0.42–0.58), with both the BD I and BD II groups demonstrating lower performance compared to the control group. In contrast, no statistically significant differences were found among the groups in automatic speech tasks (Months and Days) (*p* > 0.05).

**Figure 1:**
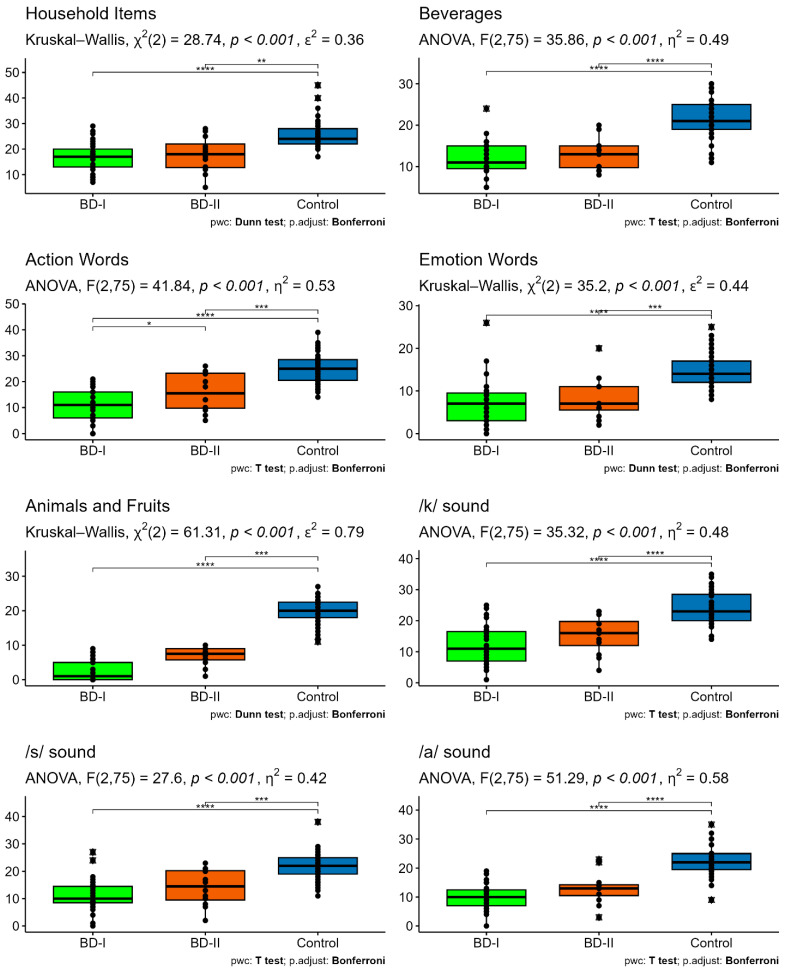
Verbal fluency differences among BD I, BD II, and control groups (* *p* < 0.05, ** *p* < 0.01, *** *p* < 0.001; **** *p* < 0.0001).

The results of the Bonferroni-corrected post hoc analyses are presented in Table 5 and Figure 1. For most verbal fluency skills, both the BD I and BD II groups differed significantly from the control group, whereas no significant differences were generally observed between the BD I and BD II groups.

Specifically, in semantic fluency tasks (Household Items, Emotion Words, and Animals and Fruits), the performances of the BD I and BD II groups were comparable, while both groups demonstrated significantly lower performance than the control group (*p* < 0.01). A similar pattern was also observed in phonemic fluency tasks (/k/, /s/, and /a/ sounds) as well as in the Beverages and Action Words categories.

A limited but statistically significant difference between the BD I and BD II groups was observed only in the Action Words category (adjusted *p* = 0.048). No significant differences were detected between the bipolar subtypes for the remaining measures. For the Months and Days categories, none of the pairwise comparisons remained significant after correction for multiple comparisons.

## 4. Discussion

In this study, the verbal fluency performance of individuals diagnosed with BD was compared both with a healthy control group and between the BD I and BD II subgroups. Statistically significant differences were found between the control and BD groups in verbal fluency tasks. The control group demonstrated superior performance across all tasks. This finding contrasts with the results of De Almeida Rocca et al. [26], who reported that the control group exhibited lower performance compared to BD patients. De Almeida Rocca et al. [26] attributed the poorer performance of the control group in verbal fluency tasks to participants’ tendency to prioritize generating more words within a limited time, thereby overlooking the rules of the task.

On the other hand, some studies have shown that individuals with BD experience difficulties in verbal fluency tasks compared to healthy controls [27], while others have reported no significant differences between the two groups [28]. According to the findings of the present study, the lower performance of the BD group can be explained by studies linking verbal fluency difficulties to mood disorders [29,30]. Furthermore, Johnston et al. [31] demonstrated that abnormalities in the organization of semantic memory may contribute to the emergence of delusions, unusual thoughts, and difficulties in speech and communication. This supports the interpretation that the semantic fluency deficits observed in individuals with BD in our study may underlie the communication challenges they experience. It should be noted that Johnston et al.’s study did not include a clinical BD sample and focused on psychosis-like semantic processing abnormalities, suggesting that the semantic fluency deficits observed in our BD participants may reflect both BD-specific and more general semantic processing disturbances.

When patients with BD I and BD II were compared, it was determined that patients with BD I exhibited lower performance in the verbs category than those with BD II. When evaluated within the framework of the literature, these findings can be associated with clinical and cognitive differences. Nwulia et al. [18] demonstrated that there are clinical, cognitive, and genetic differences between patients diagnosed with BD I and BD II. Since the cognitive domain also encompasses verbal memory and executive functions, it requires well-structured semantic network connections [32]. It has been reported that patients diagnosed with BD I tend to have a less organized semantic structure compared to individuals diagnosed with BD II [29]. In the present study, the lower performance of patients with BD I in the verbs category compared to patients with BD II supports these findings.

The superior performance of patients with BD II in the verbs category may also be related to their encoding and retrieval abilities. Chang et al. [29] demonstrated in their study on semantic memory in BD I and BD II patients that BD I patients, compared to both BD II patients and healthy controls, relied less on conventional strategies such as the encoding and retrieval of information based on its general features in semantic categorization. Consistent with these findings, the results of the present study also indicate that participants with BD II and the healthy control group exhibited higher performance in the verbs category compared to participants with BD I. This is an expected finding considering the severity of the disorder and its cognitive–linguistic effects. In other words, the strategies employed by BD I patients in semantic memory-related classifications involve processing less information than those used by BD II patients.

Cognitive impairments observed in bipolar disorder, including deficits in attention, working memory, inhibition, and executive functions, can mechanistically influence verbal fluency performance [33]. Semantic and phonemic fluency tasks require strategic word retrieval and production processes, which rely heavily on the functional integrity of prefrontal executive systems; impairment in these systems in BD may therefore lead to reduced verbal output [34]. These deficits are not limited to laboratory measures but may also affect daily functioning, manifesting as delays in social communication, decreased performance in academic tasks, and difficulties in interpersonal interactions [35]. In this context, the relationship between cognitive and language impairments represents a key mechanism influencing everyday functional outcomes in individuals with BD. The findings of the present study align with the existing literature on the impact of executive dysfunction on verbal fluency and its implications for social and academic functioning, providing a theoretical framework for future research [33].

Clinical studies and meta-analyses indicate various cognitive impairments in BD (particularly in attention, memory, and executive functions) [36,37,38,39]. As Burgess [40] suggested, executive functions encompass a wide range of cognitive processes and behavioral abilities, such as problem solving, sequencing, verbal reasoning, cognitive flexibility, the ability to sustain attention, planning, resistance to interference, utilization of feedback, multitasking, learning, and coping with novelty. Inhibition, which is related to executive function skills, has been particularly studied in semantic fluency tasks [41]. De Almeida Rocca et al. [26] demonstrated that individuals with BD experience difficulties in executive function skills such as initiation and inhibition. In the semantic switching task of this study, participants were asked to produce words alternately and sequentially from the categories of animals and fruits. Since switching between categories also requires the ability to inhibit semantic information, this resulted in lower performance among patients with BD.

Phonemic fluency skills require multiple cognitive abilities, including working memory and self-regulation. As noted by Frangou et al. [42], phonemic verbal fluency tasks depend on frontal lobe function, particularly the dorsolateral prefrontal cortex; therefore, the phonemic fluency difficulties observed in patients with BD may be associated with frontal dysfunctions present in this disorder.

Based on the study’s quantitative data, participants’ productivity varied across tasks. Individual and group-level differences in task performance may not always reflect an underlying impairment in the semantic system [17]. Reduced category fluency in BD may indicate a disruption of semantic knowledge, but it may also reflect merely a degradation of conceptual representations [43]. Conversely, observing normal productivity in BD does not necessarily imply that the semantic system is intact. In other words, impaired productivity may indicate some deterioration in semantic knowledge, whereas unimpaired productivity does not necessarily indicate an intact semantic system, as certain semantic deficits may not reduce task performance [43]. In this regard, it is important to analyze verbal fluency data not only quantitatively but also through content analysis.

In the study, no significant differences were observed between groups in automatic word categories, such as days and months. In contrast, impairments were observed in semantic and phonemic fluency. The preservation of automatic speech is likely due to these productions being supported by distinct cognitive and neural mechanisms. Van Lancker Sidtis and Sidtis (2018) reported that formulaic expressions are associated with the right hemisphere and subcortical structures [44], while Marko et al. (2023) indicated that automatic speech activates different brain regions compared to other types of language production [45]. In the study by Birn et al. (2010), bilateral superior temporal and precentral gyrus activations were observed during automatic speech [46]. These regions support the storage of overlearned and frequently used word sequences and the processes of sequential retrieval. On the other hand, semantic and phonemic fluency require executive processes, including word search, strategic retrieval, and inhibition, which are mediated by prefrontal regions such as the left inferior frontal gyrus and dorsolateral prefrontal cortex [47]. Therefore, while automatic speech tasks can be maintained relatively independently of prefrontal executive systems, semantic and phonemic fluency rely on prefrontal executive mechanisms that are affected in bipolar disorder, resulting in observed impairments.

### Limitations and Future Research Directions

This study has several limitations that should be acknowledged. First, the relatively small sample size limits the statistical power to detect potential differences, particularly between bipolar disorder subtypes. Future studies with larger samples would contribute to a more robust and reliable examination of cognitive and linguistic differences across these subtypes. Second, although key demographic variables such as age, sex, education level, and socioeconomic status were taken into account, data regarding participants’ occupational status were limited. This restriction precluded a comprehensive evaluation of the potential effects of occupational functioning and cognitive demands on verbal fluency performance. Future research incorporating more detailed occupational and functional demographic variables may help clarify the determinants of verbal fluency abilities in individuals with bipolar disorder. Third, all participants with bipolar disorder were recruited from a single psychiatric clinic located in Istanbul. This may limit the generalizability of the findings to patients from different geographical regions, treatment settings, or cultural contexts. Accordingly, future multicenter and cross-cultural studies are warranted to enhance the external validity of the results. Fourth, the types, dosages, treatment continuity, and medication adherence related to psychotropic drug use were not examined in detail. Given that psychotropic medications may exert differential—and sometimes opposing—effects on cognitive functioning, pharmacological treatment variables represent an important potential confounding factor in the assessment of verbal fluency performance. Future studies are therefore encouraged to systematically evaluate medication-related variables. Fifth, although illness duration and number of episodes were included in the analyses, the clinical severity of the disorder was not directly assessed using standardized measures. Incorporating indicators such as symptom severity, functional impairment, and need for clinical intervention in future studies would allow for a clearer understanding of how verbal fluency performance relates to the current clinical burden of the disorder. Sixth, information regarding participants’ most recent hospitalizations and the time elapsed since the last acute episode was not examined in detail. Considering that proximity to acute episodes and recent hospitalizations may influence cognitive and language performance, inclusion of these variables in future research may facilitate a more accurate clinical interpretation of verbal fluency outcomes. Finally, this study did not include comprehensive cognitive or neuropsychological assessments that would allow for the disentanglement of the underlying cognitive processes contributing to verbal fluency performance. Future studies incorporating detailed cognitive and neuropsychological evaluations may provide a more integrative understanding of the relationship between verbal fluency abilities and general cognitive functioning in bipolar disorder.

## 5. Conclusions

This study demonstrated statistically significant differences between the control group and the bipolar disorder (BD) groups in verbal fluency tasks, with the control group exhibiting superior performance across all tasks. When patients with BD I and BD II were compared, the BD II group was found to perform better in the verbs category than in the other subcategories. These findings indicate that verbal fluency skills vary according to the type of BD. The results of this study are particularly important for identifying the verbal fluency skills of patients with BD whose native language is Turkish, revealing differences between BD I and BD II, and determining distinctions among verbal fluency subcategories. For future research in this field, the use of comparative study designs between groups is recommended. The significant difference observed between BD I and BD II in the verbs category, in particular, may provide important insights into whether the type and severity of the disorder differentially affect neuropsychological functions related to these tasks and whether this task is more sensitive in distinguishing between the two groups. Further studies examining linguistic, cognitive, and neuropsychological variables using larger samples and methods such as ROC analysis are expected to make substantial contributions to the field.

## Figures and Tables

**Table 1:** Inter-rater reliability (ICC[2,1]) of verbal fluency tasks under blinded scoring conditions.

	ICC (Single Measures)	95% CI (Lower)	95% CI (Upper)
Household items	0.913	0.909	0.915
Beverages	0.914	0.911	0.916
Action words	0.915	0.913	0.917
Emotion words	0.91	0.905	0.913
Animals and fruits	0.915	0.913	0.916
/k/ sound	0.914	0.911	0.916
/s/ sound	0.913	0.909	0.915
/a/ sound	0.914	0.911	0.916
Months	0.854	0.820	0.877
Days	0.897	0.800	0.965

ICC values were calculated using a two-way random-effects model with absolute agreement (ICC[2,1]).

**Table 2:** Effect of diagnostic group on verbal fluency measurements, controlled for age (ANCOVA).

	Effect	df	F	*p*	η^2^p
Household items	Bipolar Type	2, 74	20.362	<0.001	0.355
	Age	1, 74	0.012	0.915	<0.001
Beverages	Bipolar Type	2, 74	35.264	<0.001	0.488
	Age	1, 74	0.003	0.958	<0.001
Action words	Bipolar Type	2, 74	40.555	<0.001	0.523
	Age	1, 74	0.072	0.789	<0.001
Emotion words	Bipolar Type	2, 74	23.473	<0.001	0.388
	Age	1, 74	0.008	0.928	<0.001
Animals and fruits	Bipolar Type	2, 74	213.141	<0.001	0.852
	Age	1, 74	0.030	0.862	<0.001
/k/ sound	Bipolar Type	2, 74	35.922	<0.001	0.493
	Age	1, 74	1.137	0.290	0.015
/s/ sound	Bipolar Type	2, 74	27.180	<0.001	0.424
	Age	1, 74	0.058	0.811	<0.001
/a/ sound	Bipolar Type	2, 74	51.741	<0.001	0.583
	Age	1, 74	0.961	0.330	0.013
Months	Bipolar Type	2, 74	1.607	0.207	0.042
	Age	1, 74	1.277	0.262	0.017
Days	Bipolar Type	2, 74	1.440	0.243	0.037
	Age	1, 74	1.455	0.232	0.019

η^2^p = Partial eta squared (Effect Size).

**Table 3:** Age-restricted (25–55 years) sensitivity analyses for verbal fluency measures.

Variable	BD-I	BD-II	Control	χ^2^	df	*p*	ε^2^
Household items	17.5 (7–29)	20 (16–25)	25 (17–45)	22.62	2	<0.001	0.424
Beverages	12 (5–24)	10 (8–15)	23.5 (12–30)	28.62	2	<0.001	0.541
Action verbs	11.5 (0–21)	13 (7–23)	26.5 (17–39)	32.98	2	<0.001	0.627
Emotion words	7 (0–26)	7 (3–13)	15.5 (8–25)	25.56	2	<0.001	0.482
Animals and fruits	0.5 (0–9)	6 (1–8)	20 (12–27)	40.47	2	<0.001	0.774
/k/ sound	10.5 (1–25)	16 (4–23)	24.5 (14–35)	27.66	2	<0.001	0.523
/s/ sound	10 (0–27)	13 (2–20)	22 (13–38)	26.39	2	<0.001	0.498
/a/ sound	9.5 (0–19)	13 (3–23)	23.5 (14–35)	33.62	2	<0.001	0.64
Months	12 (0–12)	12 (12–12)	12 (12–12)	4.39	2	0.111	0.067
Days	7 (0–7)	7 (7–7)	7 (7–7)	2.87	2	0.237	0.037

Values are presented as median (minimum–maximum). Group differences were assessed using the Kruskal–Wallis test in the age-restricted (25–55 years) sample. BD-I = Bipolar Disorder type I; BD-II = Bipolar Disorder type II.

**Table 4:** Demographic and clinical characteristics of the patient and control groups.

		BD I (n = 27)	BD II (n = 12)	Control (n = 39)	*p*	Effect Size
Age, Md (Min–Max) ‡		43 (23–66)	25 (19–58)	41 (20–63)	0.081	0.04 ε^2^
Illness Duration (Months), M (SD)		88.81 (55.9)	76.75 (20.9)	-		
Gender, n (%) §						
Female		17 (63)	8 (66.7)	25 (64.1)	0.976	0.02 V
Male		10 (37)	4 (33.3)	14 (35.9)		
Education, n (%) §						
Primary School		4 (14.8)	3 (25)	6 (15.4)	0.871	0.14 V
Middle School		3 (11.1)	0 (0)	5 (12.8)		
High School		8 (29.6)	5 (41.7)	14 (35.9)		
University		12 (44.4)	4 (33.3)	14 (35.9)		
Socioeconomic Status, n (%) §						
Low		10 (37)	6 (50)	12 (30.8)		
Middle		12 (44.4)	4 (33.3)	17 (43.6)	0.805	0.11 V
High		5 (18.5)	2 (16.7)	10 (25.6)		
Mania, n (%)						
1		15 (56)				
2		5 (18.5)	-	-		
3		5 (18.5)	-	-		
4		2 (7.4)	-	-		
Episodes	Depression, n (%)		-	-		
0		10 (37)	0 (0)			
1		12 (44.4)	10 (83.3)	-		
2		3 (11.1)	2 (16.7)	-		
3		2 (7.5)	0 (0)	-		
Hypomania, n (%)						
1		-	8 (67)	-		
2		-	3 (25)	-		
3		-	18 (12)	-		
Household Items, Md (Min–Max) ‡		17 (7–29)	18 (5–28)	24 (17–45)	<0.001 *	0.36 ε^2^
Beverages, M (SD) †		12.19 (4.2)	12.92 (3.91)	21.49 (5.27)	<0.001 *	0.49 η^2^
Action Words, M (SD) †		10.52 (6.56)	16 (7.39)	24.97 (5.96)	<0.001 *	0.53 η^2^
Emotion Words, Md (Min–Max) ‡		7 (0–26)	7 (2–20)	14 (8–25)	<0.001 *	0.44 ε^2^
Animals and Fruits, Md (Min–Max) ‡		1 (0–9)	7.5 (1–10)	20 (11–27)	<0.001 *	0.79 ε^2^
/k/ sound, M (SD) †		11.89 (6.47)	15.33 (6.09)	23.92 (5.38)	<0.001 *	0.48 η^2^
/s/ sound, M (SD) †		11.19 (6.03)	14.08 (6.62)	21.38 (5.02)	<0.001 *	0.42 η^2^
/a/ sound, M (SD) †		10.15 (4.54)	13.08 (5.58)	22.56 (5.28)	<0.001 *	0.58 η^2^
Months, M (SD) †		11.07 (3.19)	12 (0)	12 (0)	0.125	0.05 η^2^
Days, M (SD) †		6.48 (1.86)	7 (0)	7 (0)	0.148	0.05 η^2^

Md = Median; Min = Minimum; Max = Maximum; M = Mean; SD = Standart deviation; † = One Way ANOVA test; § = Chi-Squared test; ‡ = Kruskal–Wallis H Test; η^2^ = Eta squared; ε^2^ = Epsilon squared; V = Cramer’s V; * = *p* < 0.05. Maximum possible scores for each verbal fluency task: Household Items = 30; Beverages = 50; Action Words = 25; Emotion Words = 30; Animals and Fruits = 25; /k/, /s/, /a/ sounds = 30 each.

**Table 5:** Post hoc pairwise comparisons of verbal fluency skills among BD I, BD II, and control groups.

	BD I vs. BD II	BD I vs. Control	BD II vs. Control
Household Items ‡	1.000	<0.001 *	0.003 *
Beverages †	1.000	<0.001 *	<0.001 *
Action Words †	0.048 *	<0.001 *	<0.001 *
Emotion Words ‡	1.000	<0.001 *	0.001 *
Animals and Fruits ‡	0.232	<0.001 *	<0.001 *
/k/ sound †	0.287	<0.001 *	<0.001 *
/s/ sound †	0.429	<0.001 *	0.001 *
/a/ sound †	0.302	<0.001 *	<0.001 *
Months †	0.481	0.160	1.000
Days †	0.535	0.191	1.000

† = Independent-samples *t*-tests + Bonferroni correction; ‡ = Dunn test + Bonferroni correction; * = *p*< 0.05.

## Data Availability

The data supporting the findings of this study are available upon request from the corresponding author. The data are not publicly available because they are part of an ongoing double-blind longitudinal study.
